# Update on Percutaneous Treatment for HFrEF: A Great Armamentarium for a Poor Ventricular Function

**DOI:** 10.31083/j.rcm2405128

**Published:** 2023-04-26

**Authors:** Antonio Sisinni, Matteo Casenghi, Antonio Popolo Rubbio, Andrea Berni, Francesco Bedogni, Emanuele Barbato

**Affiliations:** ^1^Clinical and Interventional Cardiology Department, IRCCS Policlinico San Donato, 20097 San Donato Milanese, Italy; ^2^Department of Clinical and Molecular Medicine, School of Medicine and Psychology, University of Rome “La Sapienza”, 1035 Rome, Italy

**Keywords:** heart failure with reduced ejection fraction, percutaneous treatment, transcatheter device, emerging technology, medical therapy

## Abstract

Pharmacological treatment is the cornerstone therapy of heart failure with reduced ejection fraction (HFrEF). In addition, several percutaneous techniques have been developed to treat symptomatic patients, with specific heart failure (HF) phenotypes (e.g., valvular heart disease) that require non-pharmacological treatment. Given their prognostic relevance, it is imperative to deliver high-level patient care. This review provides a clinical overview on the available data regarding transcatheter devices in the armamentarium of contemporary interventional cardiologists, focusing on the clinical and anatomical selection criteria.

## 1. Introduction

**Fig. 1.**
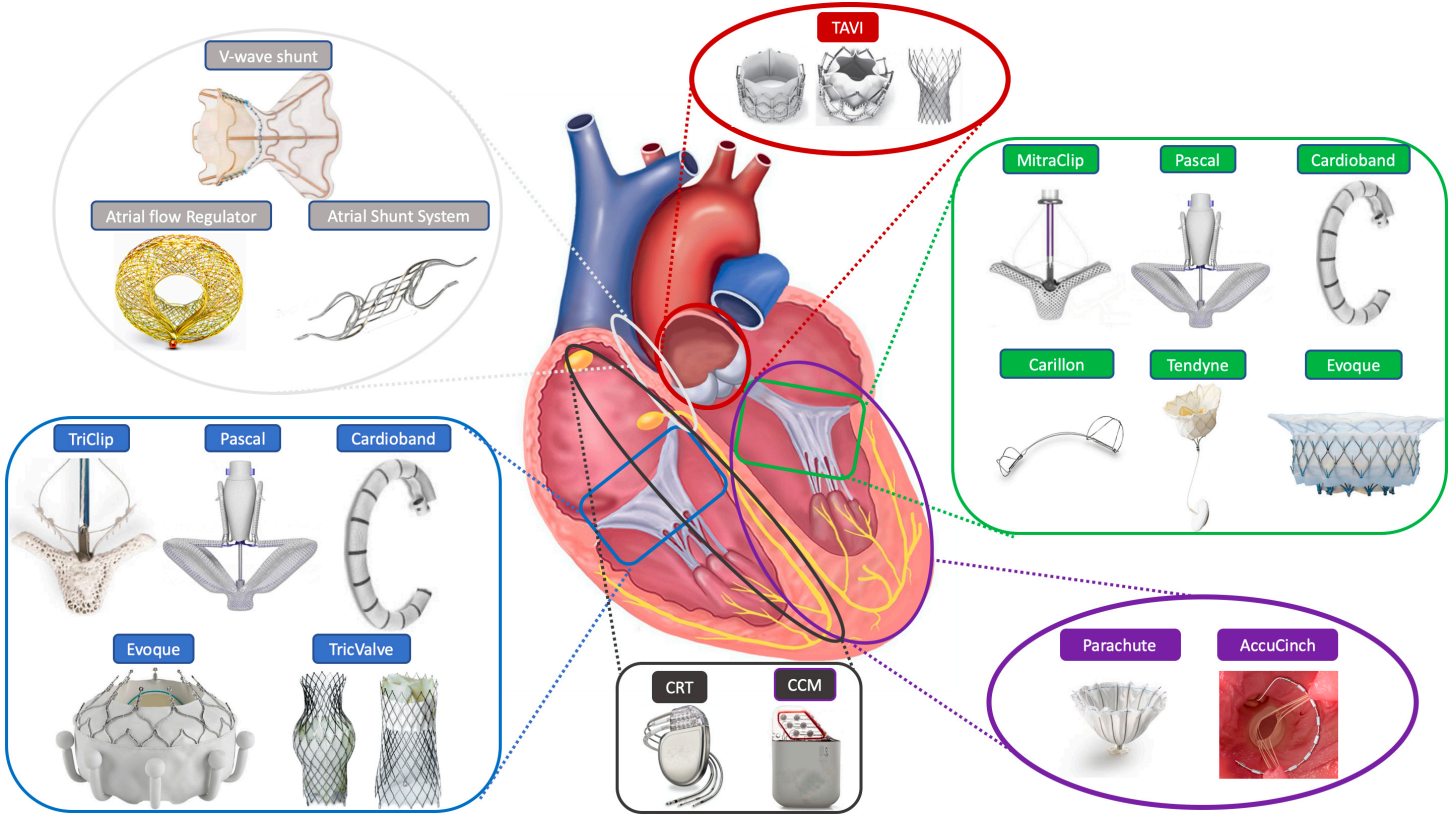
**The armamentarium of transcatheter option for HFrEF treatment**. CCM, cardiac contractility modulation; CRT, cardiac resynchronisation therapy; TAVI, transcatheter aortic valve implantation.

Heart failure (HF) is present in about 1–2% of the adult population in developed countries, with approximately a half being affected from HF with reduced ejection fraction (HFrEF), defined by the presence of symptoms and/or signs of HF and a left ventricular ejection fraction (LVEF) ≤40% [1]. Pharmacological treatment is the cornerstone therapy of HFrEF for both clinical and prognostic improvement, with the available evidence having established a multi-drug approach involving the ‘fantastic four’: beta-blockers, mineralocorticoid receptor antagonists, angiotensin converting enzyme inhibitors or angiotensin receptor/neprilysin inhibitors and sodium-glucose co-transporter 2 inhibitors [2]. Collectively, these drugs are estimated to reduce cardiovascular mortality or hospitalization for HF by 64% [3]. However, further improvement of clinical outcomes may be achieved by addressing specific underlying pathologies (e.g., valvular heart disease) that require non-pharmacological treatment in patients already on optimal medical therapy. For such reasons, several percutaneous devices have emerged in the past few years as new tools to treat symptomatic patients with HFrEF. Transcatheter treatments can be grouped according to their mechanism of action into (a) valvular replacement/repair, (b) interatrial shunt, and (c) left ventricular (LV) remodeling devices. In this review, we aim to summarize the existing data regarding the role of transcatheter devices in the treatment of HF (Fig. 1), with emphasis on the best clinical and anatomical criteria for patient selection, current recommendations for implantation (Table 1, Ref. [1, 4]), and ongoing studies aimed at expanding these indications (Table 2).

**Table 1.** **Current European Society of Cardiology indications to device treatment of patients with HFrEF**.

Device		Indication	Class of recommendation	Level of evidence
Aortic stenosis°				
	TAVI	≥75 years or STS-PROM or EuroSCORE II >8% or unsuitable for surgery	I	A
Secondary mitral regurgitation°				
	TEER	symptomatic patients, not eligible for surgery and fulfilling criteria suggesting an increased chance of responding to the treatment	IIa	B
	transcatheter annuloplasty	n.a.		
	transcatheter replacement systems	n.a.		
Concomitant aortic stenosi and secondary mitral regurgitation°				
	TAVI + TEER	TAVI followed by MV TEER (in case of persisting severe SMR) in symptomatic patients, judged not appropriate for surgery by the Heart Team	IIa	C
Tricuspid regurgitation°				
	TEER	inoperable symptomatic patients, at a Heart Valve Centre with expertise in the treatment of tricuspid valve disease	IIb	C
	transcatheter annuloplasty			
	transcatheter replacement systems			
	heterotopic caval valve			
Interatrial shunt devices*				
	V-wave Shunt	n.a.		
	Atrial Flow Regulator	n.a.		
	Transcatheter Atrial Shunt System	n.a.		
Left ventricular remodeling devices				
	Parachute device	n.a.		
	AccuCinch	n.a.		

EuroSCORE, European System for Cardiac Operative Risk Evaluation; HFrEF, heart failure with reduced ejection fraction; MV, mitral valve; SMR, secondary mitral regurgitation; TAVI, transcatheter aortic valve implantation; STS-PROM, Society of Thoracic Surgeons predicted risk of mortality; TEER, transcatheter edge-to-edge repair; n.a., not applicable. °2021 ESC/EACTS Guidelines for the management of valvular heart disease: Developed by the Task Force for the management of valvular heart disease of the European Society of Cardiology (ESC) and the European Association for Cardio-Thoracic Surgery (EACTS) [4]. *2021 ESC Guidelines for the diagnosis and treatment of acute and chronic heart failure [1].

**Table 2.** **Principal ongoing studies on percutaneous devices for the treatment of HFrEF patients**.

Device and NCT	Trial full title	Trial acronym	Trial type	Estimated enrollement (*n*)	Anticipated completion date	LVEF inclusion criteria	Study arms	Primary endpoint
**Aortic stenosis**								
TAVI								
NCT02661451	Transcatheter Aortic Valve Replacement to UNload the Left Ventricle in Patients With ADvanced Heart Failure: A Randomized Trial	TAVR UNLOAD	RCT	300	Mar-24	LVEF >20% and <50%	*Device*: TAVR (with SAPIEN 3) and GDMT; *Control*: GDMT alone	1-year all-cause death, disabling stroke, hospitalizations related to HF, symptomatic aortic valve disease or non-disabling stroke - or - clinically significant worsening of HF, change in Kansas City Cardiomyopathy Questionnaire (KCCQ) relative to baseline
**Secondary mitral regurgitation**								
MitraClip								
NCT02444338	A RandomizEd Study of tHe MitrACliP DEvice in Heart Failure Patients With Clinically Significant Functional Mitral Regurgitation	Reshape-HF2	RCT	650	Jun-24	LVEF of ≥15% to ≤35% (if in NYHA functional class II) or of ≥15% to ≤45% (if in NYHA functional class III or IV)	*Device*: MitraClip device plus GDMT; *Control*: GDMT alone	2-year composite rate of recurrent HF hospitalizations and cardiovascular death
NCT05292716	Mitral Regurgitation Treatment in Advanced Heart Failure	MITRADVANCE	RCT	172	Apr-25	LVEF ≤35%	*Experimental*: MitraClip device plus GDMT; *No Intervention*: GDMT alone	3-month Absolute change in overall KCCQ summary score
Pascal								
NCT03706833	Edwards PASCAL TrAnScatheter Valve RePair System Pivotal Clinical Trial (CLASP IID/IIF): A Prospective, Multicenter, Randomized, Controlled Pivotal Trial to Evaluate the Safety and Effectiveness of Transcatheter Mitral Valve Repair With the Edwards PASCAL Transcatheter Valve Repair System Compared to Abbott MitraClip in Patients With Mitral Regurgitation	CLASP IIF	RCT	1275*	Jan-28	n.a.	*Experimental*: PASCAL System; *Active Comparator*: Mitraclip System	Pascal non-inferiority to MitraClip with respect to MACEs at 30-day and time to first HF hospitalization or death at 5-year
Cardioband								
NCT03016975	Annular ReduCtion for Transcatheter Treatment of Insufficient Mitral ValvE (ACTIVE): A Prospective, Multicenter, Randomized, Controlled Pivotal Trial to Assess Transcatheter Mitral Valve Repair With Edwards Cardioband System and GDMT vs GDMT Alone in Patients With FMR and Heart Failure	ACTIVE	RCT	12	Sep-24	n.a.	*Experimental*: ©Edwards Cardioband System plus GDMT; *Active Comparator*: GDMT alone	Hierarchical comparison of MR ≤2+ and cardiovascular death, number of HF hospitalizations, improvement in 6-minute walk test (6MWT) distance and KCCQ
Carillon Mitral Contour System								
NCT03142152	Assessment of the Carillon Mitral Contour System in Treating Heart Failure With at Least Mild Functional Mitral Regurgitation	EMPOWER	RCT	300	Dec-28	LVEF ≤50%	*Intervention*: Carillon Mitral Contour System and GDMT; *Control*: GDMT alone	Intervention group is superior to the control group on the hierarchical composite endpoint of death, transplant or left ventricular assist device, percutaneous or surgical MV intervention, HF hospitalization, improvement in KCCQ, and improvement in 6MWT at 1-year
Tendyne								
NCT03433274	Clinical Trial to Evaluate the Safety and Effectiveness of Using the Tendyne Transcatheter Mitral Valve System for the Treatment of Symptomatic Mitral Regurgitation	SUMMIT	RCT	958	Jun-27	LVEF >25%	*Treatment*: Tendyne Transcatheter Mitral Valve System; *Control*: MitraClip system	1-year survival free of HF hospitalization
Intrepid								
NCT03242642	Transcatheter Mitral Valve Replacement With the Medtronic Intrepid TMVR System in Patients With Severe Symptomatic Mitral Regurgitation	APOLLO	prospective, non-randomized, interventional, pre-market trial	1350	Oct-28	LVEF >30%	*Device*: Intrepid Transcatheter Mitral Valve Replacement System	1-month all-cause mortality or HF hospitalization days or KCCQ improvement
**Tricuspid regurgitation**								
TriClip								
NCT03904147	Clinical Trial to Evaluate Cardiovascular Outcomes In Patients Treated With the Tricuspid Valve Repair System Pivotal	TRILUMINATE Pivotal trial	RCT	700	Dec-28	LVEF >20%	*Device*: TriClip plus GDMT; *Control*: GDMT alone	1-year hierarchical composite of number of participants with all-cause mortality or number of participants with tricuspid valve surgery, rate of HF hospitalizations, and assessment of quality of life improvement using the KCCQ
Pascal								
NCT04097145	A Prospective, Multicenter, Randomized, Controlled Pivotal Trial to Evaluate the Safety and Effectiveness of Transcatheter Tricuspid Valve Repair With the Edwards PASCAL Transcatheter Valve Repair System and Optimal Medical Therapy (OMT) Compared to OMT Alone in Patients With Tricuspid Regurgitation	CLASP II TR	RCT	825	Mar-29	n.a.	*Experimental*: PASCAL System & GDMT; *Active Comparator*: GDMT alone	2-year composite of all-cause mortality, right ventricular assist device implantation or heart transplant, TV intervention, HF hospitalizations, and quality of Life improvement (measured by KCCQ score)
**Inteatrial shunt device**								
V-wave shunt								
NCT03499236	REducing Lung congestIon Symptoms Using the v-wavE Shunt in adVancEd Heart Failure	RELIEVE-HF	RCT	605	Oct-27	n.a.	*Treatment*: V-Wave Shunt implantation plus GDMT; *Control*: GDMT alone	1- to 2-year Hierarchical composite of death, heart transplant or LVAD implantation, HF hospitalizations, worsening HF events treated as an outpatient, and change in KCCQ
**Left ventricular remodeling device**								
AccuCinch								
NCT04331769	Randomized Clinical Evaluation of the AccuCinch® Ventricular Restoration System in Patients Who Present With Symptomatic Heart Failure With Reduced Ejection Fraction (HFrEF)	CORCINCH-HF	RCT	400	Dec-30	LVEF ≥20% and ≤40%	*Device*: AccuCinch Ventricular Restoration System plus GDMT; *Control*: GDMT alone	1-year hierarchical composite endpoint of all-cause deaths, LVAD implants or heart transplants, HF hospitalizations, and changes from baseline in KCCQ Overall Score

* refers to both CLASP IID/IIF trials. CLASP IIF, Edwards PASCAL TrAnScatheter Mitral Valve RePair System Pivotal Clinical Trial; FMR, functional mitral regurgitation; GDMT, guideline-directed medical therapy; HF, heart failure; HFrEF, heart failure with reduced ejection fraction; KCCQ, Kansas City Cardiomyopathy questionnaire; LVAD, left ventricular assist device; LVEF, left ventricular ejection fraction; MACE, major adverse cardiovascular event; MITRADVANCE, Mitral Regurgitation Treatment in Advanced Heart Failure; MR, mitral regurgitation; NCT: national clinical trial; NYHA, New York Heart Association; RCT, randomized controlled trial; TAVI, transcatheter aortic valve implantation; TAVR-UNLOAD, Transcatheter Aortic Valve Replacement to UNload the Left Ventricle in Patients With ADvanced Heart Failure; n.a., not applicable.

## 2. Valvular Heart Disease Treatment Devices

### 2.1 Aortic Stenosis

The hemodynamic consequences of aortic stenosis (AS) consist in an increased LV afterload, reduced myocardial compliance due to fibrosis, and increased myocardial workload resulting into a progressive pressure-related LV remodeling [5, 6]. These changes in LV function and workload are believed to evolve into a progressive systolic and diastolic dysfunction resulting in a complex interaction between transvalvular flow, mean gradient, and LVEF.

Symptomatic severe AS has a dismal prognosis and timely intervention is strongly recommended [7]. Since the first-in-human procedure performed by Alain Cribier in 2002, transcatheter aortic valve implantation (TAVI) has shown impressive progress in terms of procedural standardization and clinical use, with its indications having been extended [8]. According to current European Society of Cardiology (ESC) guidelines, TAVI is recommended (Class of Recommendation I, Level of Evidence A) in patients aged >75 regardless of pre-operative risk estimation, as defined by Society of Thoracic Surgeons (STS) predicted risk of mortality (PROM)/European System for Cardiac Operative Risk Evaluation (EuroSCORE) II [4]. Such recommendation is based on results of randomized control trials (RCT) suggesting that TAVI is non-inferior or even superior to surgical aortic valve replacement (SAVR) in high- and intermediate-risk patients at mid-term follow-up, and in low-risk patients at short-term follow-up [9, 10, 11, 12, 13, 14]. In patients with impaired LVEF, its limited invasiveness and the associated faster recovery might provide a further advantage over cardiac surgery.

In the context of HFrEF, two different phenotypes of AS can be identified: high gradient AS (HG-AS) and low-flow low-gradient AS (LFLG-AS). Commonly, HG-AS with low LVEF is associated with an afterload-mismatch, in which the increased afterload causes a reduction of the stroke volume and a decline in ejection fraction despite a preserved contractile reserve. In such cases, the resolution of the AS might lead to an improvement in LV systolic function and a regression in LV mass hypertrophy. In a single-center 5-year analysis of cardiovascular mortality, major adverse cardiovascular and all-cause mortality, events did not differ significantly between HG-AS patients dichotomized according to baseline LVEF (preserved vs. reduced), thus demonstrating that TAVI in HG-AS patients with HFrEF had clinical outcomes similar to patients with preserved LVEF [15].

Conversely, LFLG-AS is usually associated to irreversible myocardial damage due to extensive myocardial fibrosis and/or concomitant coronary artery disease. Other conditions, such as atrial fibrillation (AF) and associated mitral and/or tricuspid regurgitation, may contribute to a low-flow state. In these settings, the magnitude of benefit achieved by valve replacement should be carefully evaluated case-by-case. Indeed, in the presence of a low transvalvular gradient at baseline or concomitant valvular disease, aortic valve replacement might be considered ineffective from an haemodynamic and prognostic standpoint [16]. On the other hand, conservative management of these patients has been associated with mortality rates >50% at 3-year follow-up while early post-operative mortality after SAVR reach up to 20% [17, 18]. Thus, although current evidence is based only on observational studies, TAVI might be considered the optimal therapeutic option for patients with severe LFLG-AS [19, 20]. The True or Pseudo-severe Aortic Stenosis (TOPAS-TAVI) was the first multicenter registry dedicated to this specific population. TAVI was associated with good periprocedural outcomes, with 30-day mortality of 3.8%, lower than the STS score-based expected of 7.7%. Residual paravalvular leaks (PVL) pulmonary disease and anemia were identified as independent predictors of adverse outcomes in terms of death and/or re-hospitalization for HF at 2-year follow-up. Interestingly, the absence of contractile reserve at baseline dobutamine stress echocardiography failed to predict clinical outcomes or LV systolic function changes [21]. According to these studies [19, 20, 21], the prevention of procedural-related factors that might further impair LVEF, such as PVL, patient-prostheses mismatch, and permanent pacemaker implantation, relies on meticulous pre-procedural planning and prosthesis selection.

Recently, Jean *et al*. [22] evaluated prognostic contribution of moderate aortic stenosis (aortic valve area 1.0 to 1.5 ${\mathrm{cm}}^{2}$, peak transvalvular velocity 2 to 4 m/s at rest or after dobutamine stress echo) in patients with LVEF <50% compared to patients with LVEF <50% and no AS. They found that, after 3-year follow-up, moderate AS was associated with an increased risk of mortality (hazard ratio [HR]: 2.98; 95% confidence interval [CI]: 2.08–4.31; *p <* 0.0001) and of the composite of mortality and HF hospitalization (HR: 2.34; 95% CI: 1.72–3.21; *p <* 0.0001). Of note, TAVI, but not SAVR, was associated with improved survival (HR: 0.43; 95% CI: 0.18–1.00; *p* = 0.05) [22].

As procedural safety of TAVI has improved, we should re-evaluate the established indications for aortic valve replacement, as we should also consider implementing its use to treat patients with moderate AS and low (¡50%) LVEF. The potential benefit of TAVI in this setting will be evaluated in the ongoing Transcatheter Aortic Valve Replacement to UNload the Left Ventricle in Patients With ADvanced Heart Failure (TAVR-UNLOAD) trial (NCT02661451) which is recruiting patients with HF, LVEF between 20% and 50%, and moderate AS to receive either TAVI on top of guideline-directed medical therapy (GDMT) or appropriate HF therapy alone.

### 2.2 Mitral Regurgitation

*Chronic setting*—Secondary (or functional) mitral regurgitation (SMR) is a common finding in patients with HFrEF [23], resulting from spherical remodeling and enlargement of the LV leading to geometrical distortion of the subvalvular apparatus. With disease progression and worsening SMR, increased leaflets tethering and decreased closing forces perpetuate the vicious cycle involved in “valvular HFrEF” until the final phase of “advanced HFrEF” characterized by recurrent acute HF episodes. Any kind of intervention at this point may be effective only in alleviating symptoms while percutaneous mitral valve (MV) interventions may reduce the burden of volume overload, thus reversing LV dilation and dysfunction and ultimately improving symptoms and even survival [24], if performed at an earlier stage.

**Transcatheter edge-to-edge repair (TEER) devices** have been developed as percutaneous counterparts of the surgical Alfieri stitch [25], in order to treat symptomatic patients with severe mitral regurgitation (MR) considered by the Heart Team at high risk for surgery or inoperable [24].

The **MitraClip system** (©Abbott Vascular, Santa Clara, CA, USA) is currently the only device whose efficacy on outcome has been evaluated in RCTs. Although it was originally conceived for the treatment of primary MR (especially considering the results of the pivotal Endovascular Valve Edge-to-Edge REpair Study (EVEREST) trial), the indication was then extended for SMR treatment too, with favorable early and mid-term results in multi-center experiences [26, 27, 28]. In reference to these studies, although limited to their intrinsic observational nature, several predictors of cardiovascular outcomes have been proposed. Among them, LVEF has expectedly shown to be a strong predictor of outcome, with lower values being associated with poorer results [29]. In addition, the etiology of MR itself and acute post-procedural results have also been shown to be associated with poorer outcome [30, 31]. In this regard, newer generations of the device, such as the MitraClip G4, allowed more satisfactory results, in terms of residual MR, even in the most complex anatomies, including extreme tethering in SMR [32]. Moreover, in patients with advanced HFrEF, intra-procedural challenges related to ventricular dysfunction, hypoperfusion, or risk of mismatch should be considered. The use of inotropic support during the procedure, as well as mechanical assist devices, can be useful in selected cases or in hemodynamic unstable patients [33].

Recent evidence from the latest RCTs has therefore highlighted the importance of an adequate patient selection and a correct intervention timing, prior to LVEF deterioration. The Cardiovascular Outcomes Assessment of the MitraClip Percutaneous Therapy for Heart Failure Patients with Functional Mitral Regurgitation (COAPT) trial showed that in patients with HF and severe SMR, TEER with MitraClip improved survival compared with GDMT alone (HR: 0.62; 95% CI: 0.47–0.82; *p <* 0.001), up to 3-year follow-up [34]. Conversely, the Percutaneous Repair with the MitraClip Device for Severe Functional/Secondary Mitral Regurgitation (MITRA-FR) trial showed neutral results in patients treated with TEER vs. GDMT [35]. Comparing the two studies is complex since MITRA-FR patients showed more severe LV dilation/dysfunction and less severe MR, suggesting that HF was, in large part, related to LV disease rather than to the valvular involvement. According to current guidelines [4], TEER should be considered (Class of Recommendation IIa, Level of Evidence B) in selected patients who are unsuitable for surgery and who fulfill COAPT trial selection criteria, as they have a higher probability of treatment response (Table 3, Ref. [34]).

**Table 3.** **COAPT-like profile suggesting an increased chance of responding to MV TEER [34]**.

All of the following criteria should be fulfilled		
Absence of left ventricular impairment	Absence of right ventricular impairment	Absence of hemodynamic instability
∙ LVEF ≥20%	∙ Tricuspid annular plane systolic excursion ≥15 mm or S’ wave velocity at tissue Doppler imaging ≥8 cm/s	∙ no HF refractory to GDMT
∙ LV end-systolic diameter ≤70 mm	∙ absence of severe TR	∙ no need for intravenous inotropes or mechanical circulatory support
	∙ systolic pulmonary artery pressure ≤70 mmHg	

COAPT, Cardiovascular Outcomes Assessment of the Mitral Percutaneous Therapy for Heart Failure Patients with Functional Mitral Regurgitation; GDMT, guideline-directed medical therapy; HF, heart failure; LV, left ventricle; LVEF, left ventricular ejection fraction; MV, mitral valve; TEER, transcatheter edge-to-edge repair; TR, tricuspid regurgitation.

Based on these trials, the definitions of proportionate and disproportionate MR were then suggested, in order to define the balance between LV dysfunction and MR degree [36]. However, sub-analysis from Mitra-FR and COAPT trials showed that the benefit of TEER with the MitraClip system is not fully supported by the proportionate-disproportionate hypothesis [37, 38]. In addition, while the baseline medical therapy in the COAPT trial had to be optimized in order to make the patient eligible, the baseline medical therapy in the MITRA-FR trial was not optimized in all patients and multiple adjustments occurred during follow-up, possibly masking the effect of TEER on outcomes. Finally, the more sustained efficacy of the MitraClip procedure found in the COAPT trial may be a consequence of a more aggressive strategy for MR correction [39].

Further insight will come from the results of the A RandomizEd Study of tHe MitrACliP DEvice in Heart Failure Patients With Clinically Significant Functional Mitral Regurgitation (Reshape-HF2) trial (NCT02444338), which has the same inclusion criteria as the COAPT trial in terms of MR severity and intermediate criteria between COAPT and MITRA-FR in terms of LV dysfunction severity, and the Mitral Regurgitation Treatment in Advanced Heart Failure (MITRADVANCE) trial (NCT05292716), which has the objective to evaluate the absolute changes in overall quality of life in patients with advanced HFrEF randomized between MitraClip therapy added to GDMT or GDMT alone.

The more recent **Pascal device** (©Edwards Lifesciences, Irvine, CA, USA) received CE mark approval for the treatment of SMR in February 2019 [40, 41]. Analysis from compassionate use experience (52% SMR patients) and early feasibility study (55% SMR patients) demonstrated Pascal to be safe and effective in the treatment of this subset of patients [42, 43]. Those results were confirmed by the expanded 1- and 2-year Edwards PASCAL Transcatheter Mitral Valve Repair System Study (CLASP) experience [44, 45]. The only available comparison between MitraClip and Pascal devices is a 2:1 propensity-matched retrospective analysis, which revealed no significant differences in terms of Mitral Valve Academy Research Consortium technical, device, and procedural success and clinical improvement at 1-year follow-up, except for higher rates of patients with MR <1+ and aborted device implantations due to an elevated transmitral gradient in PASCAL-treated group [46]. Head-to-head comparison trials such as the Edwards PASCAL TrAnScatheter Mitral Valve RePair System Pivotal Clinical Trial (CLASP IIF) (NCT03706833) will provide further data.

**Transcatheter annuloplasty** implants, such as **Cardioband** (©Edwards Lifesciences, Irvine, CA, USA) and **Carillon Mitral Contour System** (©Cardiac Dimensions, Kirkland, Washington DC, USA), were designed to reduce MV annulus to minimize regurgitation. The Cardioband Mitral System is a transcatheter, transseptal, adjustable, direct mitral annuloplasty device. In a multicentre study, the Cardioband mitral system demonstrated safety and reasonable performance (MR ≤2+ in ~ 60% of patients) at 1-year follow-up. Notably, significant restriction of posterior leaflet mobility due to extreme or highly asymmetric tenting or close proximity of the left circumflex coronary artery to the planned location of device anchors were anatomies considered ineligible for Cardioband implantation [47]. The Annular ReduCtion for Transcatheter Treatment of Insufficient Mitral ValvE (ACTIVE) trial (NCT03016975) will evaluate the Cardioband system in conjunction with GDMT compared to the former alone in this subset of patients. The Carillon Mitral Contour System is a right-heart indirect MV annuloplasty device exploiting the close relationship of the coronary sinus (CS) to the mitral apparatus. Limitations of the Carillon System are (a) the need for a suitable distance between the CS and the mitral annulus, (b) the risk of distal anchor-related compression on the left circumflex coronary artery and (c) its contraindicated use in patients with a pacing lead in the CS [47]. A comprehensive, individual patient data meta-analysis demonstrated that Carillon-based annuloplasty provided clinically significant benefits in terms of New York Heart Association (NYHA) functional status, left atrium (LA) and LV volumes, and MV performance indexes in patients with HF and SMR [48]. The ongoing Assessment of the Carillon Mitral Contour System in Treating Heart Failure With at Least Mild Functional Mitral Regurgitation (EMPOWER) trial (NCT03142152) is randomizing patients to undergo either the Carillon implant procedure or an index procedure similar to the intervention group but without device placement (to ensure that patients will not be able to deduce the group assignment). One-year freedom from major adverse events is then going to be compared in both groups. Despite these transcatheter mitral annuloplasty technologies having received approval several years ago, none of these devices have seen the same widespread use in daily clinical practice as TEER techniques [49].

Lastly, **transcatheter replacement** systems such as **Tendyne **(©Abbott Vascular, Santa Clara, CA, USA) and **Intrepid** (©Medtronic, Minneapolis, MN, USA) are emerging therapies that address the clinical need of MR treatment in the context of unfavorable anatomies for TEER or annuloplasty, including short, calcified, or severely tethered leaflets, severe annular calcification, multiple jets of regurgitation, and elevated transvalvular gradient [50].

The Tendyne is a 36-F transapical MV replacement device. In the Expanded Clinical Study of the Tendyne Mitral Valve System, its impact on MR severity and symptomatic improvement was evaluated for a 2-year follow-up period in a cohort of 100 patients (89% SMR) undergoing Tendyne implantation. The overall mortality rate was 39%, the highest in the first 3 months after procedure together with re-hospitalization for HF rate. Among survivors, a statistically significant reduction of LVEF was noticed, despite the presence of hemodynamic improvement in terms of pulmonary pressure reduction [51]. However, potential complications associated with transapical delivery, such as myocardial injury, bleeding, thoracotomy incisional pain, ventricular arrhythmias and prolonged hospitalization, remain an important aspect to consider, especially in frail elderly patients with reduced LVEF [52]. The Clinical Trial to Evaluate the Safety and Effectiveness of Using the Tendyne Transcatheter Mitral Valve System for the Treatment of Symptomatic Mitral Regurgitation (SUMMIT) trial (NCT03433274) will provide the opportunity to evaluate the safety and clinical benefits of Tendyne compared to the MitraClip System in patients with LVEF >25% within approved MitraClip indications or in patients with MV disease due to severe mitral annular calcification.

The Intrepid is a 35-F transfemoral, transseptal MV replacement device whose safety and efficacy at 30-day follow-up have been evaluated in a series of 15 patients (33% SMR), revealing excellent valve function and no mortality or stroke [53]. Transseptal MV replacement remains a challenging approach for numerous reasons, including excessive access-site bleeding (related to sheath size and need for anticoagulation to minimize the risk for valve thrombosis), safety of transseptal access, ability to align with the mitral annulus, and the need to obtain adequate height above the MV in order to minimize the risk for LV outflow tract obstruction while achieving optimal valve position [54]. The Transcatheter Mitral Valve Replacement with the Medtronic Intrepid™ TMVR System in Patients with Severe Symptomatic Mitral Regurgitation (APOLLO) trial (NCT03242642) will evaluate this valve in a larger cohort in a longer follow-up and assess LV remodeling related to elimination of MR, in patients with LVEF >30%.

*Acute setting*—Ischemic SMR may emerge as a consequence of papillary muscle rupture or apical and inferior displacement of the papillary muscles due to rapid remodeling of the infarcted area. Acute SMR can lead to acute pulmonary edema and hemodynamic instability, affecting short-term prognosis. The efficacy of TEER using the MitraClip system in patients with acute SMR has been retrospectively evaluated and compared to conservative and surgical treatment [55]. Of note, patients with papillary muscle rupture were excluded from analysis. Patients undergoing conservative strategy had the worst prognosis whereas survival was higher in TEER-treatment group when compared to surgery, mainly due to lower in-hospital mortality rate. Hence, authors have suggested that TEER may be considered a rescue therapy for patients that are deemed high risk for surgical intervention. However, it should be taken into account that neither randomized clinical trials nor other analyses comparing percutaneous treatment of MR with other clinical strategies in this acute and complex setting are currently available.

### 2.3 Tricuspid Regurgitation

In the context of HFrEF, increased left atrial pressure (LAP) may lead to (a) increasing post-capillary pulmonary pressure, resulting in pulmonary hypertension (PH), right ventricle (RV)-to-pulmonary artery (PA) uncoupling and, finally, RV dilation and dysfunction and (b) LA negative remodeling and enlargement with subsequent atrial AF onset, volume overload, and progressive right atrium (RA) enlargement. All these mechanisms may elicit tricuspid annulus (TA) dilation leading to leaflet malcoaptation and, ultimately, tricuspid regurgitation (TR). Furthermore, lead-induced TR has to be considered [56, 57]. In the last few years, percutaneous transcatheter tricuspid repair/replacement systems emerged as a new tool to treat symptomatic high surgical risk patients with severe TR. Transcatheter devices can be grouped into leaflet devices for TEER, annuloplasty devices, transcatheter TV replacement and heterotopic caval valve implantation. Global RV dysfunction is a predictor of outcomes among patients undergoing percutaneous procedures [58].

**Leaflet devices**, whose target is to improve leaflet coaptation, are the most commonly applied for interventional TR treatment, as they combine the possibility to repair rather than replace with the experience acquired on MV treatment through a transcatheter approach [59]. The Trial to Evaluate Treatment With Abbott Transcatheter Clip Repair System in Patients With Moderate or Greater Tricuspid Regurgitation (TRILUMINATE) enrolled 85 patients with moderate or greater TR undergoing **TriClip **(©Abbott Vascular, Santa Clara, CA, USA) implantation in a prospective, multicenter, single-arm study. After 1 year of follow-up, a durable reduction to moderate or less TR in 71% of patients, a significant clinical benefit in terms of NYHA functional class, and a reduction of the rate of rehospitalization by 40% compared to the period before the procedure was reported [60]. Non-anteroseptal location of the TR and a coaptation gap >8.5 mm were identified as predictors of procedural failure [61]. Similarly, the Edwards PASCAL TrAnScatheter Valve RePair System in Tricuspid Regurgitation (CLASP TR) study reported significant TR improvement to no more than moderate in 52% of patients treated with the **PASCAL device** (©Edwards Lifesciences, Irvine, CA, USA), with excellent safety profile at 30-day follow-up [62]. TriClip and Pascal devices are being studied in pivotal randomized controlled trials compared to medical therapy (TRILUMINATE Pivotal Trial [NCT03904147] and CLASP II TR trial [NCT04097145], respectively) and will address whether TR reduction improves clinical outcomes.

In the TriBAND study, the **Cardioband direct annuloplasty system** (©Edwards Lifesciences, Irvine, CA, USA) showed to be effective in reducing significantly the septo-lateral diameter, leading to an improvement of TR severity to moderate or less in 69% of patients at 30 days follow-up [63]. One of the main complications related to device implantation is right coronary artery perforation or occlusion, which occurred in 15% of the cases.

The **Evoque bioprosthesis** (©Edwards Lifesciences, Irvine, CA, USA) is delivered by a 28-F transfemoral system. The prospective, single-arm, multi-centre Edwards EVOQUE Tricuspid Valve Replacement: Investigation of Safety and Clinical Efficacy after Replacement of Tricuspid Valve with Transcatheter Device (TRISCEND) study demonstrated technical feasibility, acceptable safety (with severe bleeding as the most frequent complication), significant TR reduction, and symptomatic improvement at 30 days [64].

Lastly, heterotopic caval valve implantation can relieve TR-related symptoms, improving venous congestion, without directly treating tricuspid valve. The **TricValve** (©P+F Products+Features GmbH, Wessling, Germany) consists of two valves implanted separately in the superior and inferior vena cava, able to treat patients with diameters of the inferior vena cava up to 40–43 mm, in the presence of a distance from the right atrium junction to the hepatic veins of at least 10 mm [61]. Six-month outcomes analysis from the Safety and Efficacy of the TricValve Transcatheter Bicaval Valves System in the Superior and Inferior Vena Cava in Patients With Severe Tricuspid Regurgitation (TRICUS EURO) study revealed high procedural success rate and significant improvements in both quality of life and functional classification. Compared to other transcatheter tricuspid repair or replacement techniques, both volume overload of the right ventricle and N-terminal pro–brain natriuretic peptide concentration increased during follow-up after TricValve implantation [65]. Heterotopic caval valve implantation might therefore represent a simplistic approach to the complex issue of tricuspid valve, and, unless longer follow-up becomes available, the procedure should be limited to patients unsuitable for tricuspid valve repair or replacement (i.e., large coaptation gap, lead-related severe TR, failed previous transcatheter valve repair, severe RV dysfunction, and too large annular size).

### 2.4 Concomitant Valvular Heart Diseases

MR frequently coexists in patients with severe AS. Despite patients with both valvular heart diseases being more compromised when compared to those without, whether concomitant MR independently affects outcomes in patients undergoing TAVI remains a matter of debate [66]. In the presence of simultaneous severe AS and secondary MR in patients suffering from HFrEF undergoing TAVI, different strategies may be adopted: (1) TAVI only, (2) combined bivalvular transcatheter therapy or (3) isolated TAVI and reassessment for possible staged MV procedure. According to current ESC guidelines, although bivalvular transcatheter procedures have been demonstrated to be technically feasible and safe, TAVI followed possibly by MV TEER (in case of persisting severe SMR) should be considered only symptomatic patients judged as unsuitable for surgery by the Heart Team [4, 67].

Moderate or severe TR is present in about one third of the patients undergoing surgical or transcatheter MV interventions, and has been proven to negatively impact prognosis and quality of life [68, 69]. Since TR does not regress after successful treatment of the MV in a majority of the patients, either concomitant or staged combined procedures should be considered based on patient anatomic and hemodynamic characteristics [70].

## 3. Atrial Flow Regulator Devices

Increase in LAP is the key determinant of pulmonary congestion, with consequent dyspnea and exercise limitation, in patients with HFrEF [71]. Moreover, it is the precipitating mechanism of acute decompensation in chronic HF [72]. An implantable device for real-time indirect monitoring of LAP (**CardioMEMS HF System**, ©Abbott Vascular, Santa Clara, CA, USA) has been shown to decrease re-hospitalization for HF by guiding dose titration of decongestion therapies [73, 74]. Similarly, devices able to reduce LAP through an interatrial communication that determines a left-right shunt have been developed. Shunt flow is based on the interatrial pressure gradient, leading to an on-demand, auto-regulating reduction in LAP [75].

While the **InterAtrial Shunt Device** (©Corvia Medical, Tewksbury, MA, USA) has been evaluated in patients with HF with preserved or at least mildly reduced LVEF, **V-wave Shunt device** (©V-Wave Ltd, Caesarea, Israel) was the first interatrial shunt technology implanted in a patient with HFrEF [76, 77]. It is a self-expanding, hourglass-shaped, percutaneously implanted device containing a one-way bioprosthetic valve implanted through the femoral vein and subsequent interatrial septal puncture. A single-arm open-label study of 38 HF patients (~79% HFrEF) with NYHA functional class III or IV on optimal medical therapy was performed at 6 centers, with the shunt device having been successfully implanted in all cases. At 1-year follow-up, significant clinical improvement was observed despite attenuation of shunt patency in 50% of patients, perhaps due to intra-shunt valve deterioration. Patients with full patent shunts exhibited significant improvements in hemodynamic parameters and had the tendency to maintain clinical benefit for a longer follow-up period [78]. The 2nd generation device, eliminating the one-way valve component, is under evaluation in patients with advanced HF, regardless of LVEF, in the ongoing Reducing Lung Congestion Symptoms using the V-Wave Shunt in Advanced Heart Failure (RELIEVE-HF) trial (NCT03499236).

The **Atrial Flow Regulator** (©Occlutech, Istanbul, Turkey) is a double disc device designed to allow interatrial bidirectional flow. The Prospective, Non-randomized, Pilot Study to Assess Safety and Efficacy of a Novel Atrial Flow Regulator (AFR) in Heart Failure Patients With Reduced Ejection Fraction or in Heart Failure Patients With Preserved Ejection Fraction (PRELIEVE) was a prospective, non-randomized, open-label, multicentre study in symptomatic HF patients (~45% HFrEF) with increased pulmonary capillary wedge pressure (PCWP; ≥15 mmHg at rest or 25 mmHg during exercise). Shunt patency with unidirectional left–right shunting was proven to be useful in all patients, with some of them experiencing symptomatic improvement [79]. To the best of our knowledge, no randomized trial is currently ongoing.

It should be considered that patients undergoing shunting procedures may suffer from right heart volume overload related to (a) increasing right atrial pressure and consequent persistent right-to-left shunt leading to hypoxemia and systemic embolization and (b) right ventricular function worsening. In addition, preservation of the interatrial septum is essential to allow any further transseptal transcatheter interventions. Therefore, a novel percutaneous atriotomy technique, the **Transcatheter Atrial Shunt System** (©Edwards Lifesciences, Irvine, CA, USA), has been developed in order to create a LA-to-CS shunt potentially able to reduce LAP without interacting with the interatrial septum. Using the right internal jugular vein as access point, CS cannulation is followed by CS-to-LA puncture and balloon dilation of the LA in order to deploy the device and create the shunt. In the first in-human application, the procedure was demonstrated to be feasible and resulted in clinical and hemodynamic improvement [80].

## 4. Cardiac Resynchronization and Cardiac Contractility Modulation

Cardiac resynchronisation therapy (CRT), either with a defibrillator (CRT-D) or without (CRT-P), represents the only percutaneous treatment of HFrEF, proposed by current guidelines, with a Class of Recommendation I Level of Evidence A [1]. In appropriately selected patients, CRT reduces mortality and improves cardiac function [81]. However, it should be considered that its benefits do not extend to patients with normal or marginally increased (120-130 ms) QRS complex duration (~80% of HFrEF patients) [82] and that almost one-third of CRT recipients are noticed to be non-responders [83]. In this perspective, QRS width (≥130 ms, preferably >150 ms), left bundle branch block QRS morphology, and two novel markers of dyssynchrony, such as apical rocking and septal flash, have been identified as predictors of response to CRT [84].

Cardiac contractility modulation (CCM) is a percutaneous device-based therapy for HF, involving the application of non-excitatory electrical impulses to the RV septal wall during the absolute myocardial refractory period in order to influence the biology of failing myocardium in terms of LV contractility improvement and positive reverse remodelling [85]. Implantation is similar to a traditional transvenous pacemaker system, but with the use of two RV leads. A meta-analysis of 3 clinical trials involving 641 patients with HF undergoing CCM in addition to optimal medical therapy compared to GDMT alone has shown a significant, albeit somewhat modest, improvement in peak oxygen consumption, 6-minute walking test, and quality of life [86]. Nevertheless, no prospective trials evaluated effects of CCM on mortality as the primary outcome. A meta-analysis of data from randomized trials suggested that CCM did not significantly improve either overall mortality or all-cause re-hospitalizations [81]. Guidelines consider current evidence insufficient to support specific recommendations for CCM [1]. However, recommendations on its use in the treatment of HF compared to CRT have been suggested. CCM could be combined with an implanted cardiac defibrillator in patients with severe LV disfunction (LVEF 25–35%), while it could be offered as the only device-based therapeutic option for patients with a moderate LV dysfunction (LVEF 35%–45%) [87].

## 5. Left Ventricular Remodeling Devices

Despite continuous improvements in percutaneous reperfusion therapy over recent decades, myocardial injury following myocardial infarction and the subsequent LV adverse remodeling leading to HF remains a major health concern [88]. Based on previous surgical experiences aimed to reverse ventricular remodeling by excluding the infarcted akinetic region, devices have been developed to attempt LV shape restoration and to potentially improve prognosis, in combination with pharmacological therapy [89, 90].

The **Parachute device** (©Cardiokinetix, Redwood City, CA, USA) is a transaortic, umbrella-like system designed to partition off the akinetic or aneurysmatic portion of the LV while restoring the elliptical shape of the LV cavity. Decreased global wall stress and improved diastolic compliance might be the mechanisms by which the implant improves cardiac performance. Importantly, LV anatomy should be carefully evaluated through pre-procedural computed tomography since prominent trabeculation or an LV moderator band is unsuitable for device implantation [91]. In the PARACHUTE First-In-Human trial, 39 patients with NYHA functional class II–IV, dilated akinetic or dyskinetic anterior-apical wall that did not necessitate revascularization and LVEF between 15% and 40% were enrolled in a nonrandomized, prospective, multicenter study. At 3-year follow-up, there was a stable and significant reduction in LV end-diastolic volume, whereas stroke volume and LVEF were also significantly lower compared to baseline [92]. The PARACHUTE IV trial (NCT 01614652) represented the first randomized controlled trial comparing device implantation plus HF medical therapy to GDMT alone, but was terminated in June 2017 after enrolling 331 patients, due to device-related safety concerns, and it is unclear whether investigation will be continued.

The **AccuCinch** (©Ancora Heart, Santa Clara, CA, USA) is the first and only percutaneous ventricular restoration system designed to treat both HF and SMR. It is an endocardial implant deployed 1–2 cm below the mitral annulus. Through 12–16 anchors implanted over a 220° arc in the subannular space and cinched together with a cable, it may reduce the basal-to-mid LV free wall circumference, improving mitral leaflet apposition and reducing LV wall tension [93]. In the early feasibility study, 21 patients with HFrEF and SMR were scheduled to receive AccuCinch procedure. Device implantation success was 90%, with an average procedure time of 150 minutes and no device-related adverse events. At 6-month follow-up, significant reductions in MR degree and LV volumes with concomitant improvements in LVEF and clinical status were observed [93]. As suggested in a case report, since the beneficial effects are progressive over a short-term follow-up, its effectiveness, which initially depends on a mechanism that involves physically altering the size or shape of the ventricle, may be secondarily enhanced by inducing biological responses that result in progressive reverse modeling [94]. The AccuCinch System is currently under evaluation in the Randomized Clinical Evaluation of the AccuCinch Ventricular Restoration System in Patients Who Present With Symptomatic Heart Failure With Reduced Ejection Fraction (CORCINCH-HF) (NCT04331769) pivotal trial, which is a prospective, randomized, open-label, multicenter, international, clinical safety and efficacy investigation designed to enroll 400 symptomatic patients with LVEF 20–40% and LV end-diastolic diameter ≥55 mm.

## 6. Conclusions

The newer and existing techniques in the armamentarium of contemporary interventional cardiologists may allow various treatment strategies in order to achieve different targets specifically tailored for the several HFrEF phenotypes. However, despite the aforementioned studies, it should be underlined that only a few procedures and devices (i.e., TAVR, mitral edge-to-edge-repair, CRT) have been proven to significantly reduce major cardiovascular events in HF patients.

Hence, device-based therapies for HF should be considered complementary to pharmacological treatment and should thus be aimed at improving prognosis when pharmacotherapy is deemed to be insufficient. Appropriate patient selection and timely indication are essential for their proper implementation and success in clinical practice and patient care.

## Abbreviations

AS, aortic stenosis; CCM, cardiac contractility modulation; CRT, cardiac resynchronisation therapy; GDMT, guideline-directed medical therapy; EuroSCORE, European System for Cardiac Operative Risk Evaluation. HF, heart failure; HFrEF, heart failure with reduced ejection fraction; LVEF, left ventricular ejection fraction; MV: mitral valve; NYHA, New York Heart Association; RCT, randomized control trials; SMR, secondary mitral regurgitation; TAVI, transcatheter aortic valve implantation; TEER, transcatheter edge-to-edge repair; STS-PROM, Society of Thoracic Surgeons predicted risk of mortality; TR, tricuspid regurgitation.
